# Terahertz time domain spectroscopy allows contactless monitoring of grapevine water status

**DOI:** 10.3389/fpls.2015.00404

**Published:** 2015-06-02

**Authors:** Luis G. Santesteban, Inés Palacios, Carlos Miranda, Juan C. Iriarte, José B. Royo, Ramón Gonzalo

**Affiliations:** ^1^Department of Agricultural Production, Universidad Pública de NavarraPamplona, Spain; ^2^Department of Electrical and Electronic Engineering, Universidad Pública de NavarraPamplona, Spain

**Keywords:** dendrometer, phloem, THz spectrometry, *Vitis vinifera* L., water relations, xylem

## Abstract

Agriculture is the sector with the greatest water consumption, since food production is frequently based on crop irrigation. Proper irrigation management requires reliable information on plant water status, but all the plant-based methods to determine it suffer from several inconveniences, mainly caused by the necessity of destructive sampling or of alteration of the plant organ due to contact installation. The aim of this work is to test if terahertz (THz) time domain reflectance measurements made on the grapevine trunk allows contactless monitoring of plant status. The experiments were performed on a potted 14-years-old plant, using a general purpose THz emitter receiver head. Trunk THz time-domain reflection signal proved to be very sensitive to changes in plant water availability, as its pattern follows the trend of soil water content and trunk growth variations. Therefore, it could be used to contactless monitor plant water status. Apart from that, THz reflection signal was observed to respond to light conditions which, according to a specifically designed girdling experiment, was caused by changes in the phloem. This latter results opens a promising field of research for contactless monitoring of phloem activity.

## Introduction

Agriculture, and especially irrigated agriculture, is the sector with the largest consumptive water use at a global scale (Frenken and Gillet, 2012). From a technical point of view, proper irrigation management requires reliable information on plant water status to support growers to make fast and effective decisions (Naor, 2006). The most frequently used information sources for decision-making are (i) estimation of water consumption from climatic data, (ii) measurement of soil water content or availability, and (iii) measurement of plant water status, or (iv) activity (Jones, 2004), being the two latter sources the most reliable ones since the information is gathered directly from the plant.

Ideally, water status estimation methods for irrigation decision-making should be sensitive to changes in plant water status, and allow early detection of water stress that enables the grower adjusting the irrigation regime before a significant yield loss or damage occurs. Nevertheless, it should as well not imply destructive sampling, allow automation and also be easy and cheap to implement. In the last five decades, there has been a remarkable development of methods, since none of the available ones matches those requirements. For instance, leaf water potential measured with the Scholander pressure bomb (Scholander et al., 1965) is considered the reference method by plant physiologists and agronomists. This is because it provides a relatively quick, flexible and easy-to-understand estimation of plant water status, but measurements are destructive and cannot be automatized. Conversely, automatable and non-destructive methods such as sap flow or plant trunk diameter variation (TDV) measurement require relatively complicated set-ups, skillful maintenance and a relatively complex data interpretation (Braun, 1997; Fernandez and Cuevas, 2010; Vandegehuchte and Steppe, 2013). As a consequence, there is a great interest in developing new methods that enable an easy, affordable and accurate non-destructive estimation of plant water status.

The terahertz (THz) frequency band, the part of the electromagnetic spectrum between classical microwaves (3 mm, 100 GHz) and the infrared (30 μm, 10 THz), is slowly becoming more technologically relevant due to the increasing number of attractive applications which can be potentially developed, i.e., medicine (skin cancer detection, caries detection,…), security and surveillance (detection of hidden weapons or explosives, detection of gasses,…), viticulture (control of the vine state), food sector, space, and aeronautics, industrial, passive tomography imaging, and investigation on proteomics in the pharmaceutical industry. In other hand, in the last decade, terahertz time-domain spectroscopy (THz-TDS) has proven to be a very strong and accurate tool for characterizing and imaging various materials (for review, see Jepsen et al., 2011). The THz-TDS is a powerful spectroscopic technique that allows the time-resolved measurement of light-matter interaction with broadband and powerful THz pulses. Contrary to other spectroscopies, amplitude and phase information are directly retrieved in a single scan, making THz TDS a powerful tool for studying absorption and gain dynamics in many different kind of materials. The time information allows further to retrieve depth information and can be used in many different disciplines for tomographic imaging, which allows to retrieve specific information coming from inside the material.

The THz wave region exhibits an interest property for the agriculture sector; i.e., the electromagnetic waves operating in this frequency range are highly sensitive to water content, which makes them a suitable candidate for plant water status characterization (Iriarte et al., 2010; Etayo et al., 2011a,b; Iriarte et al., 2011; Maestrojuan et al., 2013). In fact, some research developed in the last decade has shown that THz-TDS transmittance correlates well with leaf water content in coffee (Jördens et al., 2009; Shakfa et al., 2009; Breitenstein et al., 2012), celery (Zhang et al., 2008), *Arabidopsis* (Castro-Camus et al., 2013), and silver fir (Born et al., 2014). The first research works were performed on detached leaves that were allowed to dry under laboratory conditions (Zhang et al., 2008; Shakfa et al., 2009), whereas later research has shown that this method could also be used *in vivo* (Breitenstein et al., 2012; Castro-Camus et al., 2013; Born et al., 2014).

However, in all the above mentioned research, THz-TDS measurements were performed in leaves, which implies a double difficulty for its implementation under field measuring conditions. On the one side, it is difficult to fix the sensors to the leaves, since holding systems may damage them or, at least, alter leaf conditions and, on the other side, the significance of the measurements made in a leave is limited, since there may be a large variability on water status between leaves in the same plant (Jones, 1990; Williams, 2012). From that point of view, and particularly for woody species, it would be more desirable to measure water status in the trunk, which would act as an integrator of plant water status. Furthermore, most of the measurements performed at leave level at THz frequencies have been performed in a transmission mode configurations, meanwhile, for trunk measurements, reflection configuration are more appropriated and therefore implemented into this paper.

The aim of this research is to evaluate for the first time the potentiality of THz-TDS reflection measurements performed on grapevine trunks to measure of plant water status non-destructively and in real time.

## Materials and Methods

### Experimental Layout

All the experiments were performed in 2013 on a 14-years-old grapevine (*Vitis vinifera* L., cv. ‘Tempranillo’) plant, which had been uprooted 2 years before from a commercial vineyard, transplanted to a 26 L pot filled with a peat:sand mix (2:1), and properly maintained outdoors. The plant was pruned as a single-cordon, and its vegetative and reproductive development was similar to that of moderate vigor field-grown plants. The experiments started on first August, when the plant had reached veraison (phenological stage 35 in Eichhorn–Lorenz scale). One month before starting the experiment the plant was transferred, for proper acclimation, to a growth chamber where all measurements took place.

During the acclimation period, the growth chamber was programmed for a 16 h photoperiod, day–night cycle, day and night temperature being, respectively, 23 and 16°C. The chamber was equipped with mixed incandescent and fluorescent lighting that provided approximately 400 μmol m^-2^ s^-1^ P.A.R (400–700 nm) at the upper part of the canopy. During the experiments, the photoperiod was 14 h day/10 h night (except when otherwise expressed), day and night temperature was fixed at 21°C to avoid temperature interferences, and the plant was irrigated back to field capacity every other day.

### Plant water Status Monitoring Methods

#### Conventional Methods

Soil water content was measured using a capacitance soil moisture sensor (mod. EC10, ECH2O, Decagon Devices Inc., Pullman, WA, USA) inserted in the pot. This sensor estimates soil volumetric moisture (m^3^ water m^-3^ soil) by determining the apparent permittivity of the soil. TDVs were also monitored using a dendrometer, that consists on a linear variable differential transformer (LVDT, mod. DF 2.5, Solartron Metrology, West Sussex, UK) fixed to the trunk by a metal frame of Invar (a metal alloy with minimal thermal expansion), that allows the detection of small changes in trunk size. TDV have been widely used to evaluate plant water status (Goldhamer and Fereres, 2001; Naor, 2006; Intrigliolo and Castel, 2007), since daily growth and the magnitude of stem contraction along a day are related to it. Water deficit reduces trunk growth, whereas the diurnal shrinking and swelling of tissues that mainly depend on the level of plant tissue hydration (Simonneau et al., 1993) and on the degree of a radial transfer of water from bark tissues into xylem or vice-versa (Parlange et al., 1975). The readings provided by the soil water probe and the dendrometer were logged every 2 min with a CR10X multiple purpose datalogger (Campbell Scientific Ltd., Logan, UT, USA).

#### Time Domain THz Reflection Characterization

**FIGURE 1:**
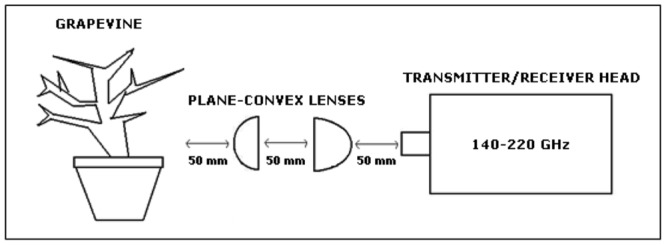
**Setup for terahertz (THz) reflection measurements**.

**FIGURE 2:**
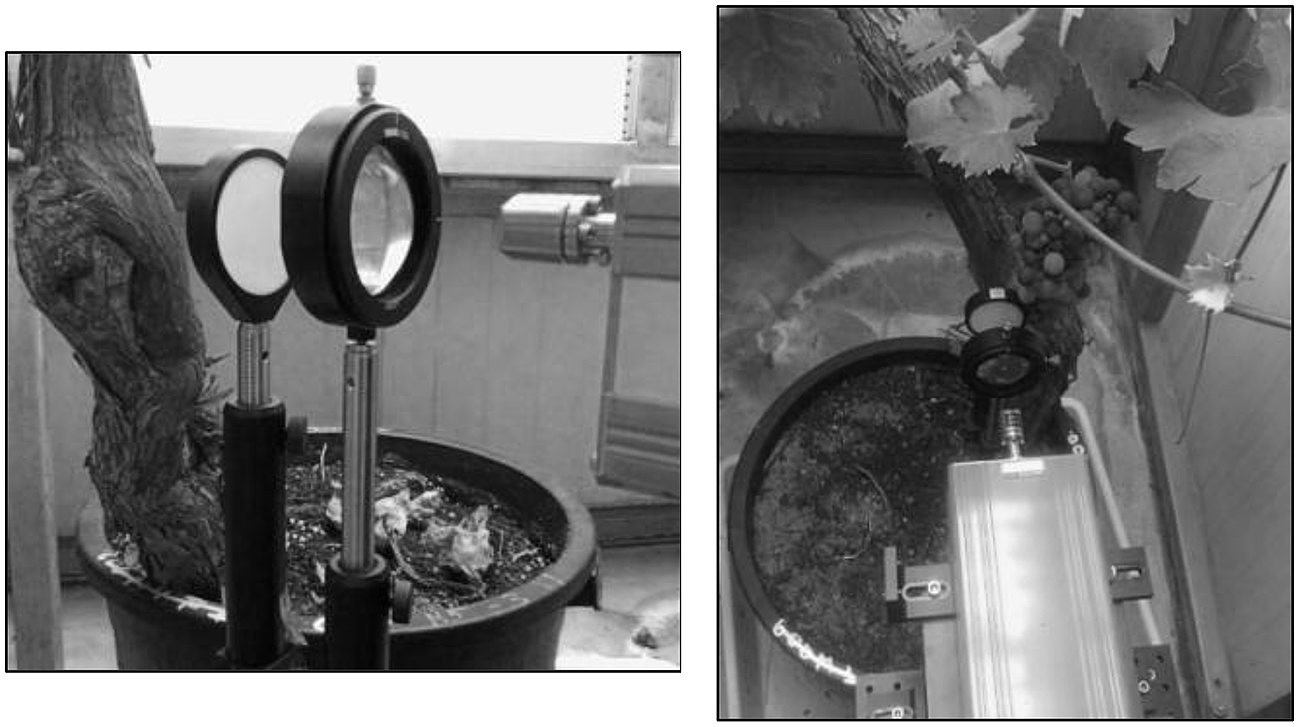
**Images of the setup for THz reflection measurements**.

Time-domain THz reflection measurements were performed on the vine’s trunk, using a network analyser (VNA, Agilent E3861C), a receiver/transmitter external frequency head in the frequency range from 140 to 220 GHz (OML V05VNA2-TR), and a pair of plane-convex lenses, whose set-up is represented in **Figure 1**, and depicted in **Figure 2**. The selected Agilent equipment is able to measure up to 26,5 GHz, but through the use of the external heads, which correspond to frequency mixers, the output operation frequency is moved to the 140–220 GHz band. The system allows to different kind of measurements; i.e., frequency and time domain responses. The VNA measures the frequency response of the device and mathematically calculates a time domain transform of the data to convert the frequency domain information into the time domain. In the reflection mode, the VNA measures reflection coefficient as a function of frequency. The reflection coefficient can be viewed as the transfer function relating the incident voltage and reflected voltage. An inverse transform converts the reflection coefficient to a function of time (the impulse response). Step and impulse responses can be calculated by convolving the input step or pulse with this reflection coefficient impulse response. The resulting measurement is a fully corrected time domain reflection response of the test device, displayed in near real-time. Response values provide valuable insight into the behavior of the grapevine trunk beyond simple frequency characteristics.

The equipment was configured to obtain automatically the time domain response of the grapevine trunk. Taking into account that the operational bandwidth is 80 GHz, the achieved spatial resolution (SR) is 1.875 mm (SR = c/2 * BW) where c is the speed of light and BW the bandwidth considered. The system recorded and processed the THz time domain reflection peak data every 10 min (real time analysis).

The plane-convex lenses are used to focus the signal from the THz receiver/transmitter external head into a smaller and concentrated beam spot over the trunk of the grapevine. This enhanced signal is achieved with a distance of 5 cm from the horn of the external frequency head to the first plane-convex lens, a distance between lenses of 50 mm, and 50 mm from the second plane-convex lens to the trunk of the grapevine. The use of these lenses improves the dynamic range of the system and prevents unwanted refractions from the trunk edges. The diameter of the beam spot achieved was of approximately 2 mm.

The underlying principle for this measurement procedure is that the reflected signal is hypothesized to be directly proportional to the water content of the grapevine in its trunk. Thus, the greater water content in the trunk, the higher refractive index is hypothesized to be encountered by the THz signal, and a higher reflection signal will be measured at the transmitter/receiver external head.

### Experiments Performed

In order to evaluate the suitability of Time Domain THz Reflection characterization for plant water status estimation, three different experiments were conducted.

#### Experiment 1: Watering Cycles

The plant was submitted to watering and drying cycles during August and the first fortnight of September. During the first 2 weeks, the plant was watered every 3–4 days leading the plant to experience mild water deficit, whereas during the following 4 weeks, watering was performed every 6–7 days, so that the plant reached a moderate to severe water deficit.

#### Experiment 2: Coupling of THz Signal to Changes in Light and Darkness

In September 23rd, the day and night pattern was changed in order to evaluate how the THz reflected signal responded to light and darkness. Thus, a light to darkness alternating cycle was programmed, consisting on 4 h-(4 h)-3 h-(2 h)-1 h-(1 h)-30′-(30′)-20′-(20′)-10′-(10′), where darkness periods are indicated between brackets.

#### Experiment 3: Coupling of THz Signal to Xylem and Phloem Activity

**FIGURE 3:**
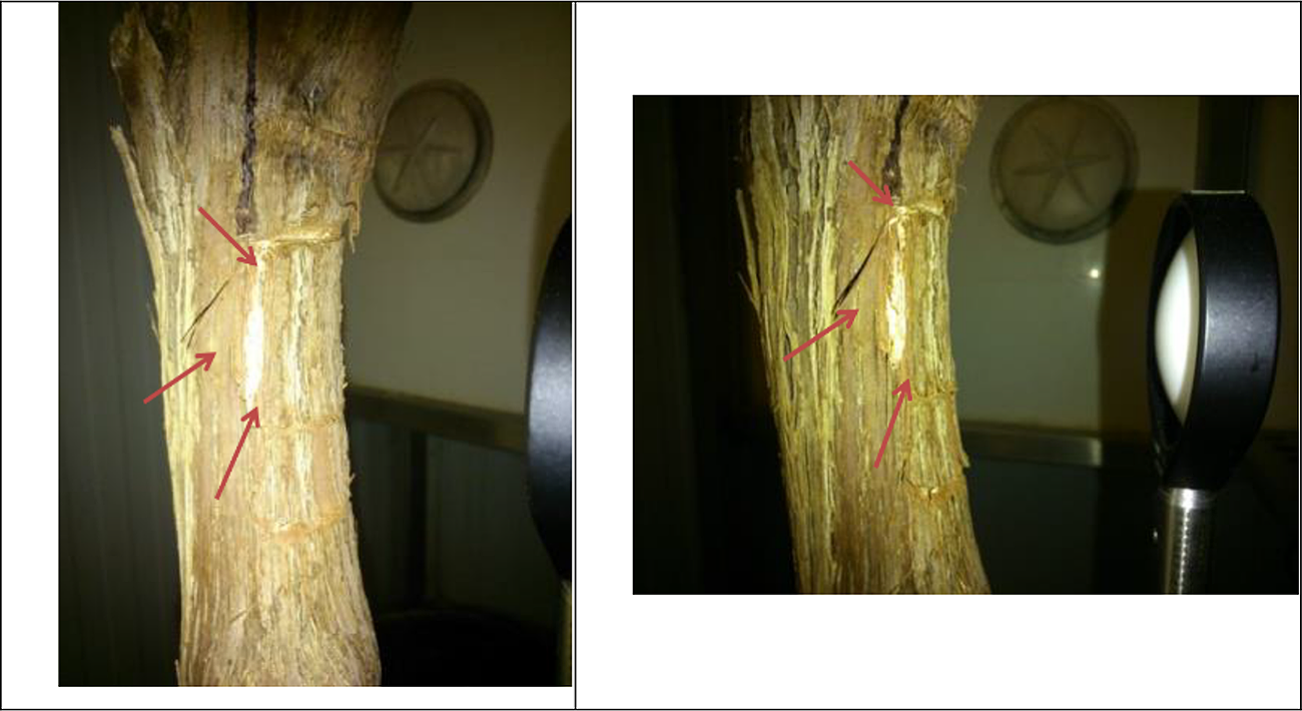
**Details of girdled area.** Phloem discontinuity lines are marked with red arrows.

In the last days of September, in order to test the dependence of the THz signal on phloem and xylem activity, phloem was discontinued in a 3 cm × 3 cm square around the lens focusing area through phloem girdling. This technique consists on removing thin strips of bark in order to prevent phloematic flux from reaching the girdled area, so that the reflected THz signal would be less dependent on phloem activity. The phloem girdled area is shown in **Figure 3**.

## Results and Discussion

### Experiment 1: Watering Cycles

**FIGURE 4:**
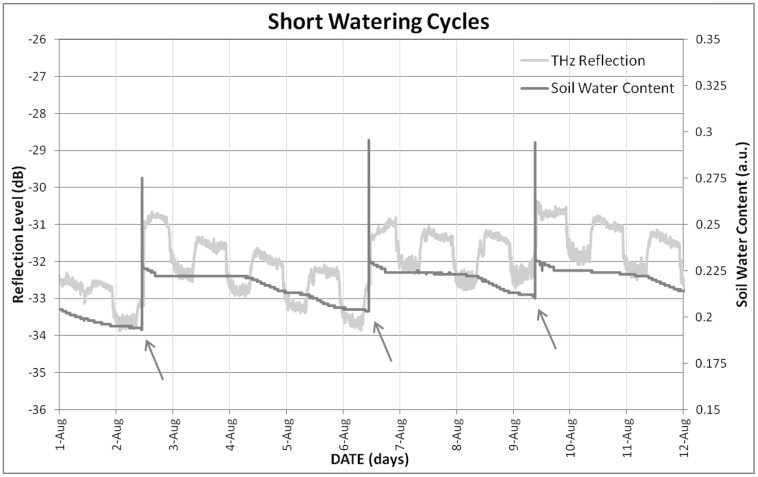
**Evolution of soil water content and trunk THz reflection signal under the short watering cycles.** Arrows indicate irrigation events.

**FIGURE 5:**
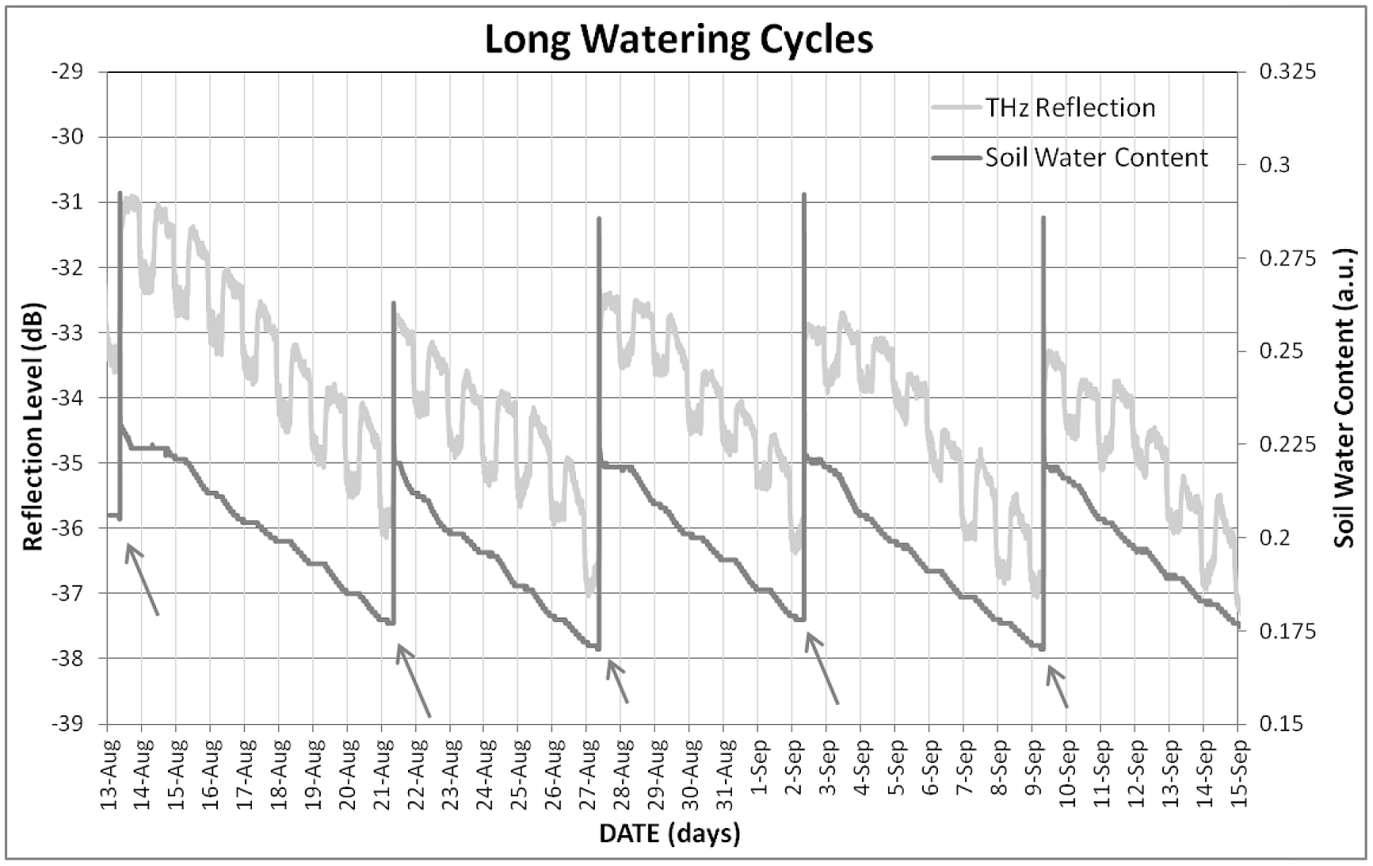
**Evolution of soil water content and trunk THz reflection signal under the long watering cycles.** Arrows indicate irrigation events.

Trunk THz time-domain reflection signal proved to be very sensitive to changes in plant water availability, as its pattern follows the trend of soil water content for either short (**Figure 4**) and long (**Figure 5**) watering cycles, the response being clearer for the latter, where a wider range of water status was embraced. THz reflection signal does not show the peak detected after every irrigation event by soil moisture sensors, which correspond to gravitational water, which gets drained in the minutes following irrigation, and is not useful for the plant. All this supports the hypothesis formulated in the introduction, linking trunk THz reflection signal to plant water status. In fact, the THz reflection signal follows the irrigation cycles by varying (increasing the amount of reflected power which means larger water content in the trunk) its level.

**FIGURE 6:**
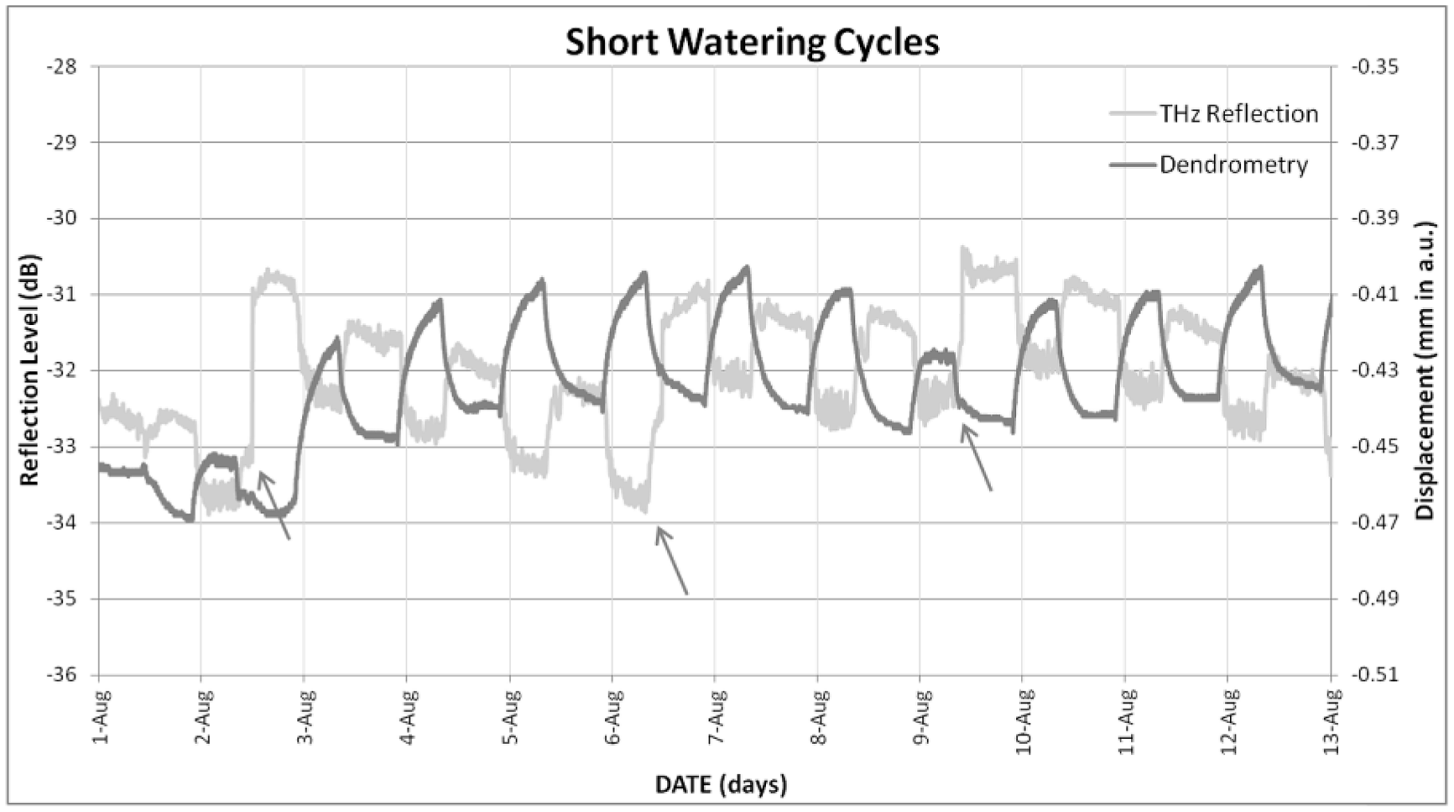
**Evolution of trunk diameter variations (TDVs) and trunk THz reflection signal under the short watering cycles.** Arrows indicate irrigation events.

**FIGURE 7:**
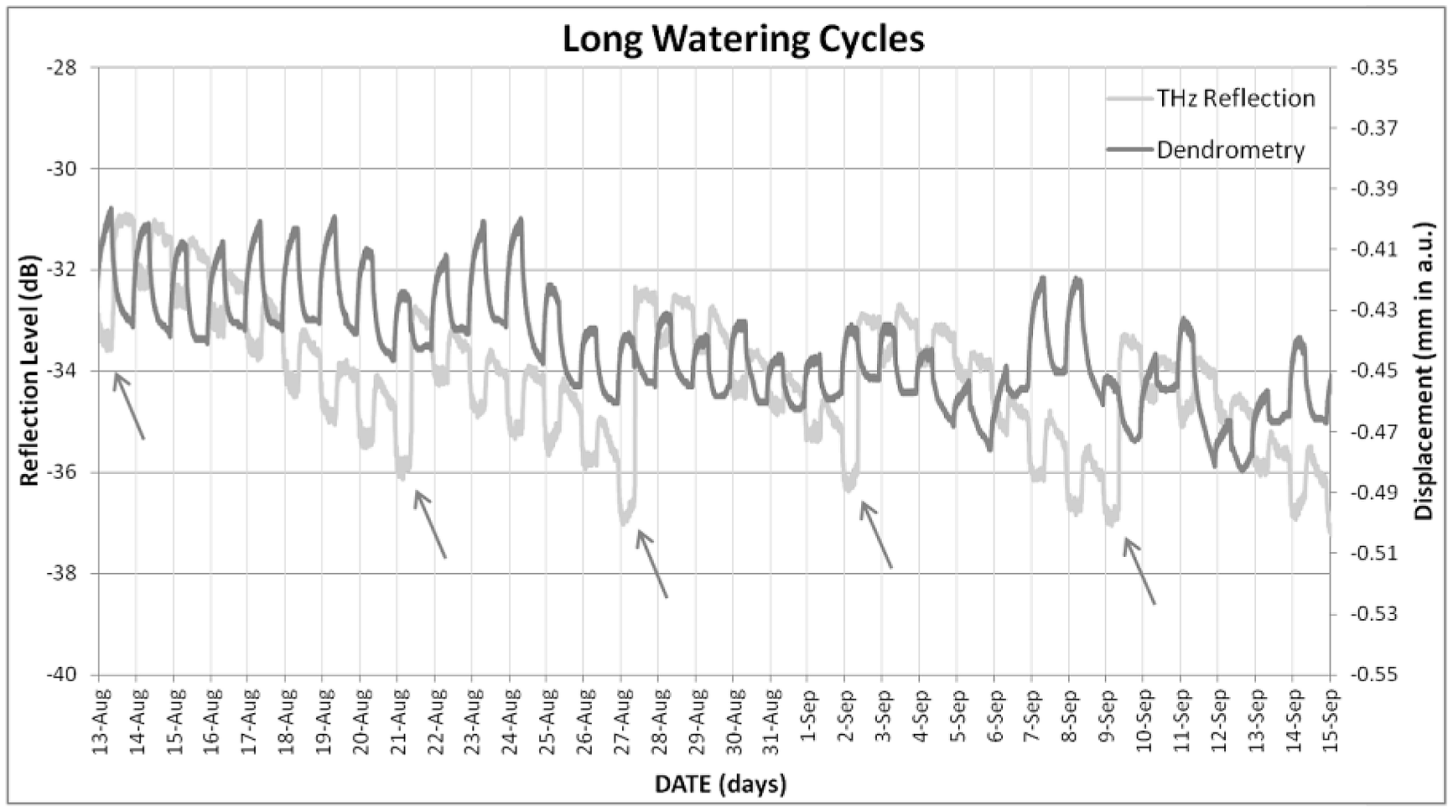
**Evolution of TDVs and trunk THz reflection signal under the long watering cycles.** Arrows indicate irrigation events.

**FIGURE 8:**
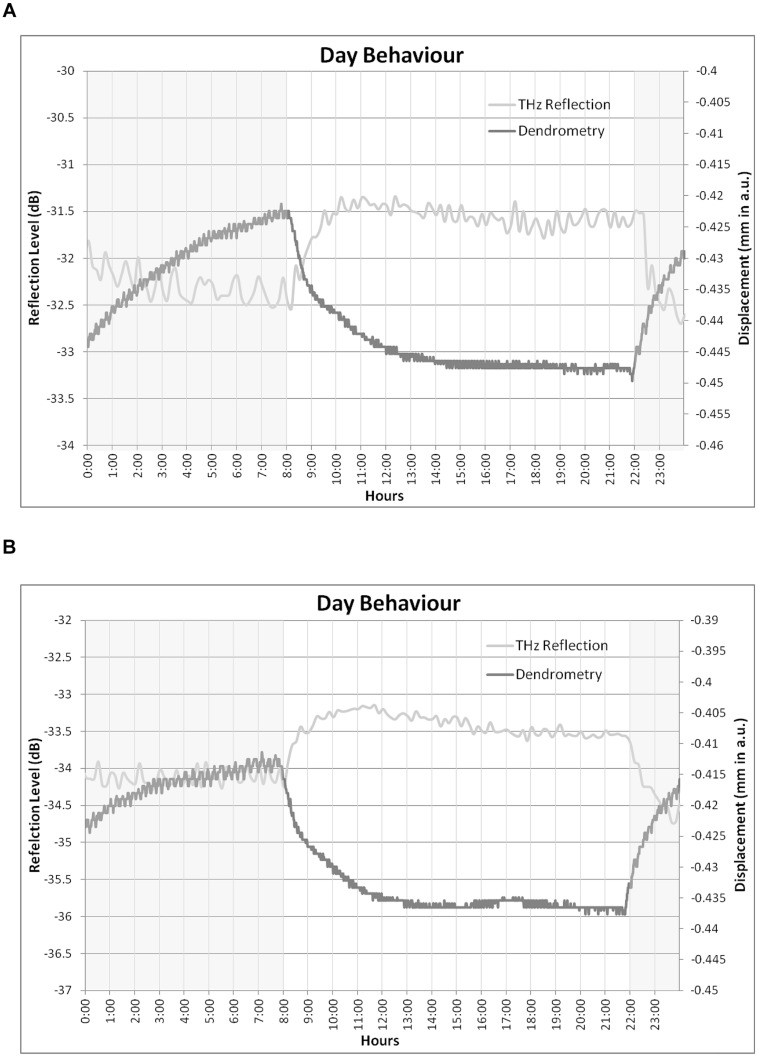
**Daily patterns for TDVs and THz reflection on **(A)** the 3rd August and on **(B)** the 22nd August**.

When THz time-domain reflection signal was compared to TDV measured with the dendrometer, the correspondence observed is not clear neither for short (**Figure 6**) nor for long (**Figure 7**) watering cycles. Taking into account the neatness of the response observed to watering events, this lack of coherence with TDV can be a consequence of the loss of sensitivity that TDV have after veraison, as shown by Intrigliolo and Castel (2007). Nevertheless, the information provided by the dendrometers was proved to be useful in order to understand the changes observed for THz-reflection signal, seen in **Figures 4–7**, the observed general tendency is quite similar, both TDV and THZ reflection measurements respond in the same way to irrigation cycles; TDV values increase when watering reducing its value between irrigation cycles, the THz reflection signal also increases when watering the plant, reducing its value between cycles. Nevertheless, if a more detailed inspection is performed at the observed ripples, these ones show that the THz reflected signal is increased during the day and decreased during the night hours. In **Figure 8**, this daily trend has been represented in more detail for 1 day in the short (**Figure 8A**) and another in the long (**Figure 8B**) watering cycles.

The THz reflection and dendrometer measurements showed opposed daily trends (**Figure 8**), so that trunk size decreases rapidly when the lights are switched on (8:00 am), whereas the THz reflection signal increases at the beginning of the day, both signals being relatively stable after the plant had been exposed to 2–4 h of daylight for the TDV measurements and 2 h for the THz reflected signal. The behavior measured by the dendrometer agrees with the expected behavior according to earlier research (see review by Fernandez and Cuevas, 2010): at the beginning of the day, stomata opened and in the first hours of light the plant gives up part of its constitutional water (initial abrupt fall) to the transpiration stream. Afterwards, nearly no variation in trunk diameters was observed, indicating balance between water loss by transpiration and uptake through the roots. Last, during the night time, since transpiration nearly ceases but water uptake from the soil is maintained, an increase of trunk diameter occurs. The inverse behavior of the THz reflection signal indicates therefore that the processes that affect it are different from those resulting in TDV. Thus, in a one day timespan, when trunk water content is known to be lower (during the day), THz reflection signal increased and trunk diameter decreased (**Figure 8**), whereas when a longer time-span (7 days) was considered (**Figure 7**), both THz reflection and diameter decreased as water availability was lower. Experiments 2 and 3 were designed in order to shed some light on this behavior.

### Experiment 2: Coupling of THz Signal to Changes in Light and Darkness

**FIGURE 9:**
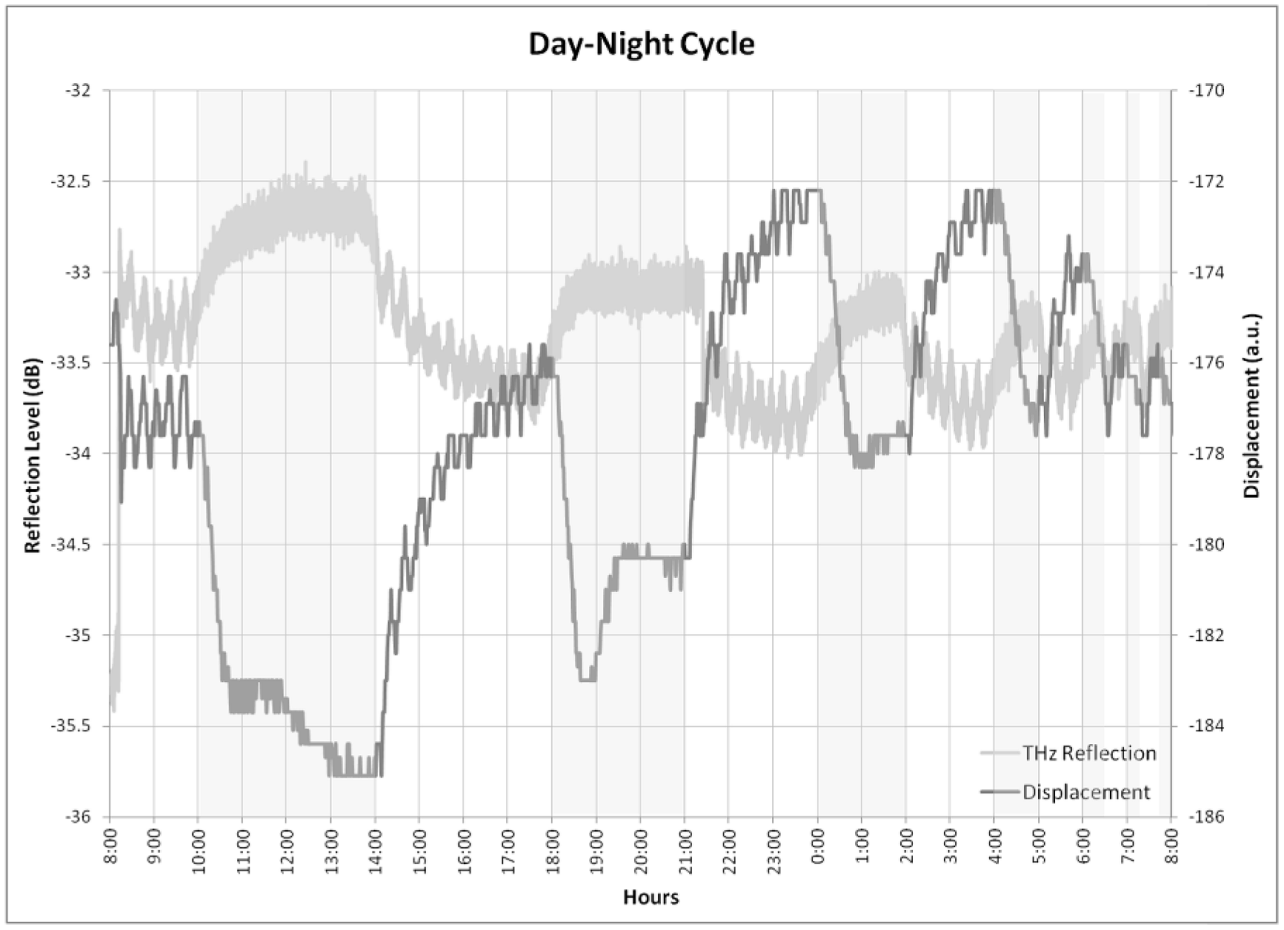
**Evolution of TDVs and trunk THz reflection under changing day–night cycles on September 23rd.** The parts of the figure with a gray background correspond to the darkness periods.

The response of THz reflection to light and darkness observed in the daily cycles in Experiment 1 was confirmed when photoperiod was changed, alternating shorter light and darkness period (**Figure 9**). When a light period started, trunk size decreased, whereas THz reflection was increased. This trend was maintained even with short light-darkness alternating periods, see the right end of the *x*-axis part of **Figure 9** (hours between 5 and 7).

Since the response of THz reflection signal to light and darkness was progressive (reflection increased gradually about 1–2 h), this behavior appears not to be a consequence of a direct response of THz reflection signal to light, but to reflect changes at a physiological level. The most obvious physiological aspect that changes between light and darkness is photosynthesis, so we hypothesized that, apart from reacting to water status in the mid-term (4–7 days), THz reflection signal was somehow responding to photosyntethic activity and/or to phloematic loading/unloading processes. In order to demonstrate this behavior, Experiment 3 was set up.

### Experiment 3: Coupling of THz Signal to Xylem and Phloem Activity

**FIGURE 10:**
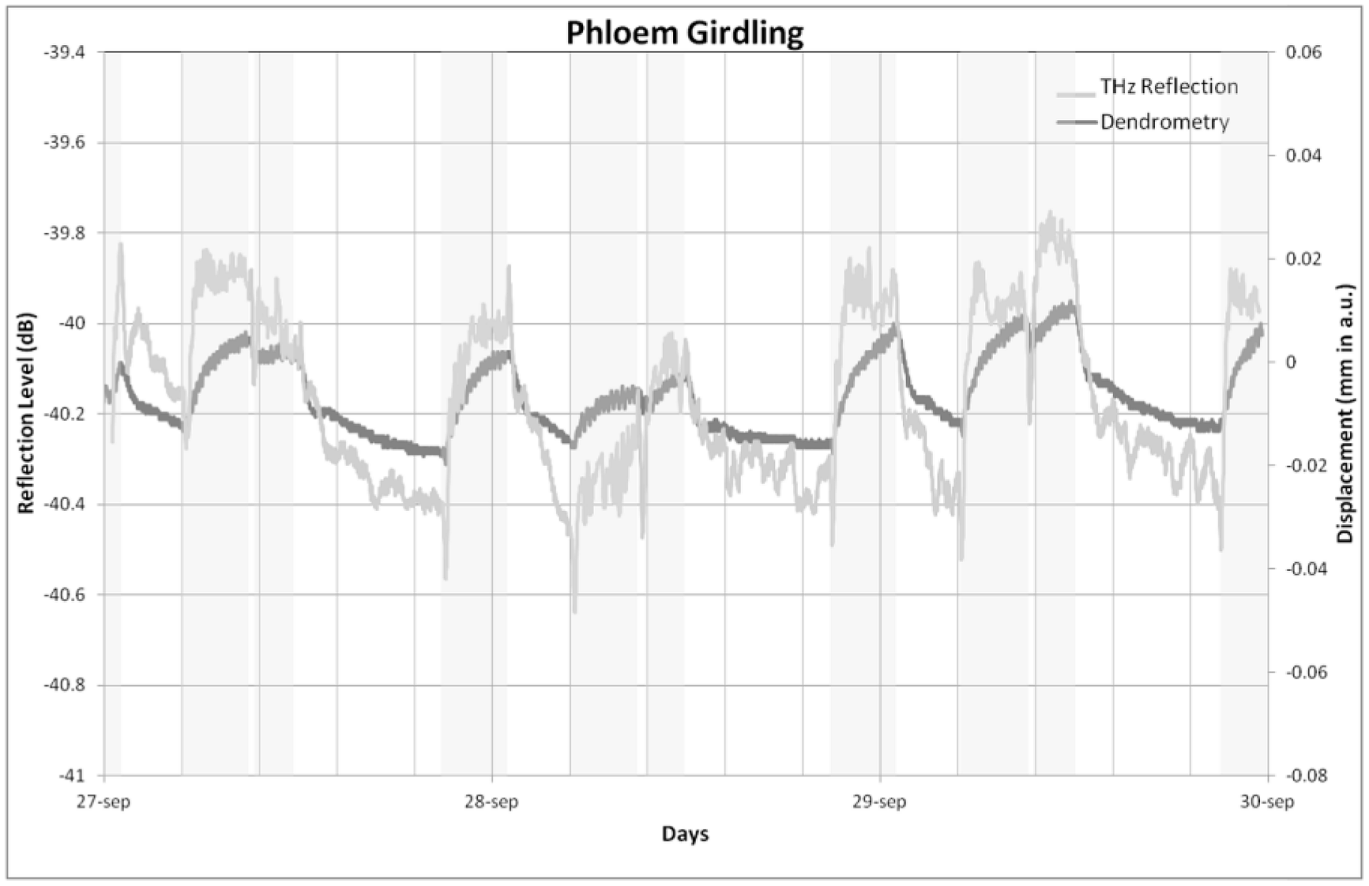
**Evolution of TDVs and trunk THz reflection under changing day–night cycles once the measurement area had been girdled.** The parts of the figure with a gray background correspond to the darkness periods.

When phloem was discontinued around the THz measurement area, the response of THz reflection to light and darkness periods was totally changed (**Figure 10**). At this point, both dendrometer and THz reflection signals follow the same trend, decreasing when during light periods, and increasing during the hours of darkness. Therefore, it appears that once phloematic flux was discontinued, THz reflection signal solely reflected the dehydration-rehydration cycles that TDV also do. On the contrary, when the plant trunk was unaltered, THz reflection was affected by xylem- and phloem-related processes, the former being preponderant in the mid-term (3–7 days) and the latter in the short term (within one day). Since THz reflection is known to depend on sugar concentration in aqueous solutions (Arikawa et al., 2008; Born and Havenith, 2009), part of these variations observed within the day may be caused by changes in phloem sugar concentration and on phloem volume, although this behavior should be studied in detail in future research.

## Conclusion

This paper has presented the potentiality of THz-TDS reflection measurements to measure the plant water status non-destructively and in real time on grapevine trunks. These results have been compared with traditional techniques based on soil moisture sensors and TDV measures with a dendrometer.

Terahertz time domain reflection measurement on trunks has been proved to be a suitable and promising tool to estimate plant water status of woody plants in real time. Moreover, the sensitivity that THz time domain reflection signal has shown to have within the day, apparently linked to phloem activity, opens another research field to be explored which can lead to new insights into the physiological activity of plants.

## Conflict of Interest Statement

The authors declare that the research was conducted in the absence of any commercial or financial relationships that could be construed as a potential conflict of interest.
